# Expression profile of microRNAs in expressed prostatic secretion of healthy men and patients with IIIA chronic prostatitis/chronic pelvic pain syndrome

**DOI:** 10.18632/oncotarget.24069

**Published:** 2018-01-06

**Authors:** Ye Chen, SuNing Chen, Jian Zhang, YangMin Wang, Zhengping Jia, Xin Zhang, Xiao Han, Xiuquan Guo, XiaoDi Sun, Chen Shao, Ji Wang, Tian Lan

**Affiliations:** ^1^ Department of Anesthesiology and Pain, Lanzhou General Hospital of Lanzhou Command, Lanzhou, China; ^2^ Department of Pharmacy, Xijing Hospital, The Fourth Military Medical University, Xi’an, China.; ^3^ Department of Biochemistry and Molecular Biology and the State Key Laboratory of Cancer Biology, The Fourth Military Medical University, Xi'an, China; ^4^ Department of Urology, Lanzhou General Hospital of Lanzhou Command, Lanzhou, China; ^5^ Key Laboratory of the Plateau of the Environmental Damage Control, Lanzhou General Hospital of Lanzhou Military Command, Lanzhou, China; ^6^ Department of Urology, Xiang’an Hospital, University of XiaMen, Xiamen, China; ^7^ Laboratory of Cell Death and Cancer Genetics, The University of Minnesota Hormel Institute, Austin, MN, United States

**Keywords:** prostate, microRNAs, chronic prostatitis/chronic pelvic pain syndrome, mir-21, high-throughput sequencing

## Abstract

The current study aimed to identify a comprehensive expression-profile of microRNAs (miRNAs) in expressed prostatic secretion (EPS) collected from healthy men and patients with CP/CPPS (Chronic prostatitis/Chronic pelvic pain syndrome). After clinical screening of 382 participants, 60 healthy men and 59 IIIA CP/CPPS patients with significant pelvic-pain were included into this study from March 2012 to December 2014. High-throughput sequencing was employed to identify characteristic expression-profile of EPS-miRNAs. QRT-PCR was further performed to confirm elevated levels of differential EPS-miRNAs. Finally, candidate EPS-miRNAs were measured traceably in 21 follow-up patients and their classify-accuracy on IIIA CP/CPPS were analyzed by ROC (receiver operating characteristic) curve. In discovery-phage, 41 and 43 predominant EPS-miRNAs were found in pooled EPS-sample from 40 healthy men and 39 IIIA CP/CPPS patients, respectively. Furthermore, 22 abundant EPS-miRNAs were up-regulated with ≥ 2-fold in 20 patients compared to 20 healthy men. In testing-phage, elevated levels of miR-21-5p, miR-30a-5p, miR-30d-5p, miR-103a-3p and miR-141-3p were further confirmed in 33 patients by comparing to 30 healthy men. In validation-phage, relieved pelvic-pain symptom of 21 follow-up patients was found to be accompanied by significant down-regulation of miR-21-5p, miR-103a-3p and miR-141-3p. Particularly, ROC curve analysis indicated the highest area under ROC curve (AUC) was found for miR-21-5p (0.891), followed in order by miR-141-3p and miR-103a-3p. Our studies provided evidence that secretory miRNAs existed in EPS and dysregulated EPS-miRNAs were associated with prostatitis. In particular, miR-21-5p possessed a high classify-accuracy for IIIA CP/CPPS patients with significant pelvic pain.

## INTRODUCTION

Organ-proximal fluid is believed to be a rich source of bio-molecules that can mirror the physiological state of a given organ, due to their high concentrations of secreted substances. Prostate is a compound tubuloalveolar organ with a specifically exocrine function to secrete a variety of substances in the semen [1, 2, 3]. Identification of the comprehensive composition of prostatic fluid is a critical step towards exploring the biological function of prostate and pathological mechanism of prostatic disease [4, 5, 6].

MicroRNAs (miRNAs) are a class of small non-coding RNA molecules sized 18–25 nucleotides which presumably regulate human genome [7]. Many important biological processes, such as cell differentiation, proliferation and apoptosis, are controlled by miRNAs [7, 8, 9]. Recently, “endocrinological function” has been attributed to miRNAs, as miRNAs are not only expressed intracellularly, but are also secreted into various body fluids [10, 11, 12, 13]. Subsequently, high throughput sequencing technology for miRNA has been developed using body fluids as a source of organ derived genetic material [14]. However, a comprehensive miRNAs sequence analysis related to prostatic fluid from healthy men and patients with prostatitis has not been reported so far.

Prostatitis is a common medical condition, which consist of four major categories:1) Category I (non-bacterial acute prostatitis); 2) Category II (bacterial chronic prostatitis); 3) Category III (chronic prostatitis/chronic pelvic pain syndrome, or CP/CPPS); and 4) Category IV (Asymptomatic inflammatory prostatitis). Category III (CP/CPPS) is the most common prostatitis syndrome encountered by urologists [15] including subgroup IIIA CP/CPPS and subgroup IIIB CP/CPPS. To provide a proof-of-concept that secreted miRNAs abundantly exist in prostatic secretion and some EPS-miRNAs may be involved in prostatic disorders, our current study focused on exploring the expression profile of EPS-miRNAs in healthy men and IIIA CP/CPPS patients.

## RESULTS

### Clinical screen of participants

**Figure 1:**
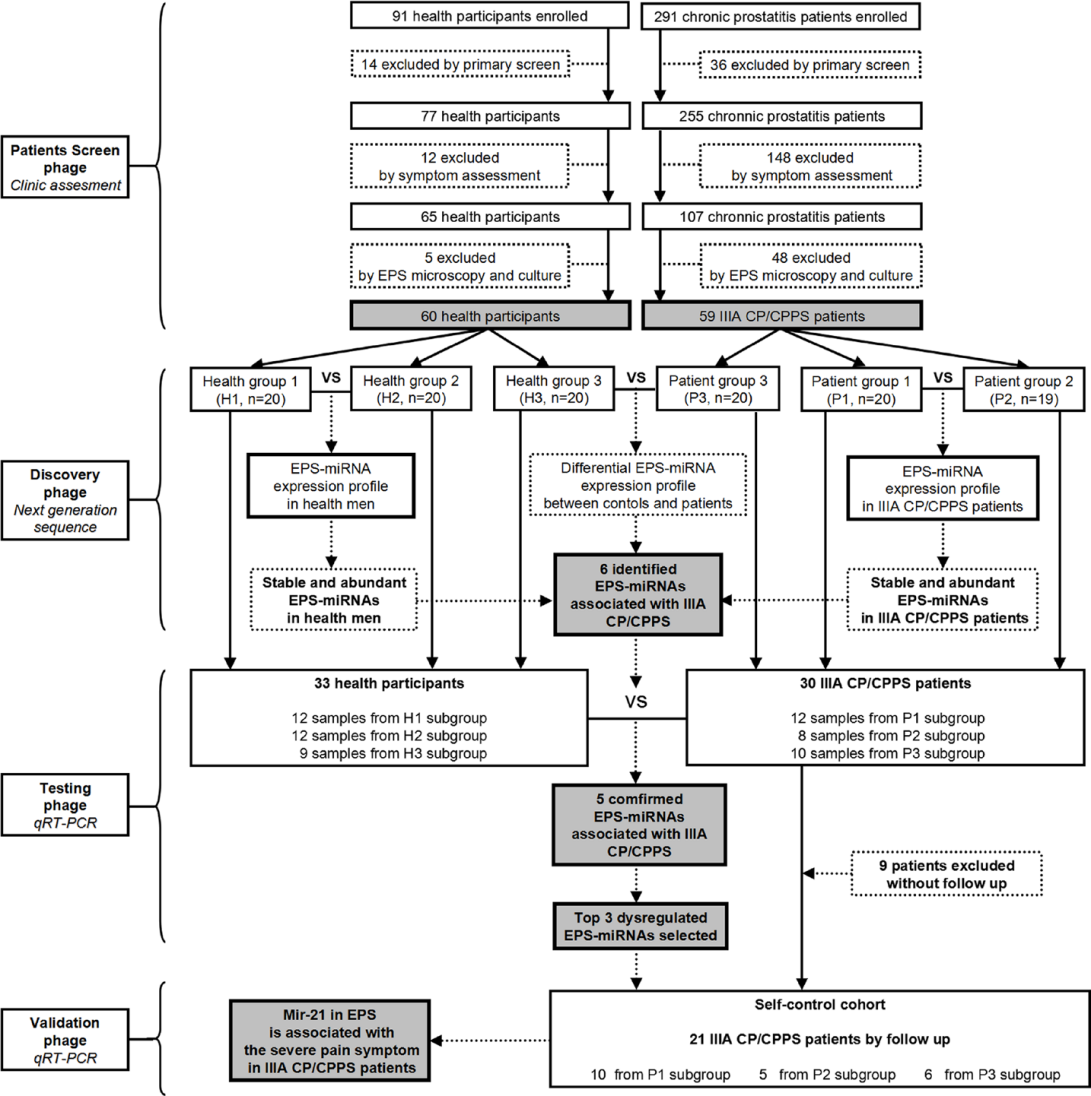
Flow diagram of participant screen and EPS-miRNA identification The procedure for identifying EPS-miRNAs consisted of four phages, including patient screen phage, discovery phage, testing phage, and validation phage. In screen phage, 60 healthy men and 59 IIIACP/CPPS patients with significant prostatitis-like pain (NIH-CPSI pain score ≥ 10) were finally included primary screen, symptom assessment, and EPS examination. In discovery phage, high-throughput sequencing was employed to identify characteristic expression-profile of EPS-miRNAs for healthy men and IIIA CP/CPPS patients. In testing phage, elevated levels of identified EPS-miRNAs were further confirmed in the individual EPS samples from 33 patients by comparing to 30 healthy men with Taqman-based qRT-PCR. In validation phage, the change levels of top dysregulated EPS-miRNAs were measured traceably in 21 follow-up patients, and their classify-accuracy on IIIA CP/CPPS were subsequently analyzed by ROC curve.

From March 2012 to December 2014, 91 healthy men underwent health examination and 291 outpatients diagnosed as chronic prostatitis (18∼45 years old) were involved in this prospective study. All subjects provided a written informed consent in accordance to the ethical guidelines of our hospital and the declaration of Helsinki. Based on the presence or absence of excessive leukocytes in EPS, CP/CPPS can be further divided into two subgroups: inflammatory IIIA CP/CPPS (WBC ≥ 10/high power field) and non-inflammatory IIIB CP/CPPS (WBC < 10/high power field) [16, 17, 18]. Of interest, an increasing body of evidence has showed that the expression profile or/and concentration of some inflammatory molecules (e.g. TNF-ɑ, IL-1 were differentially presented in prostatic secretion (EPS) from patients with IIIA and IIIB CP/CPPS, suggesting that different pathological mechanism or/and pathological process might be involved in the category IIIA and IIIB. Therefore, to screen appropriate healthy control and IIIA CP/CPPS patients with significant pelvic pain, all participants have been selected by a three-step procedure: 1) primary screen, including questionnaire investigation (to record demographic character and medical history), laboratory serum-test and ultrasound examination (to exclude other disorders), and urine-culture/urethral-swab test (to exclude infection in urinary tract), 2) symptom assessment to select patients being suffered from significant prostatitis-like pain (NIH-CPSI pain score ≥ 10) in the past week, 3) EPS microscopic examination and culture to exclude subjects with other types of prostatitis and infections rather than IIIA CP/CPPS (Table 1 and Figure 1). In clinical screen phage, all symptom assessment and EPS collection were conducted before medication to avoid the potential interference of drug usage on EPS-miRNAs expression.

**Table 1:** Inclusion and exclusion criteria for healthy men and IIIA CP/CPPS patients with significant prostatitis-like pain

	Healthy men	Patients
**Inclusion criteria**	1. Men underwent health examination in our health-check center 2. ≤ 45 years old due to the increased risk of prostate cancer or hyperplasia 3. ≥ 18 years old	1. Outpatients with chronic prostatitis diagnosed by our hospital or other clinic within 1 year
	**Primary screen:**	
**Exclusion criteria**	1. Previous surgery and permanent medication of urogenital tract (chronic prostatitis was not included)	
	2. Urogenital congenital malformation and malignancy	
	3. Neurogenic disease of the bladder	
	4. Lithiasis	
	5. Urine-culture/urethral-swab test to exclude bacterial infection and special infection (e.g. mycoplasma, Ureaplasma urealyticum, Chlamydia trachomatis, and Neisseria gonorrhoeae)	
	6. Infertility to reduce potential interference by idiopathic etiology	
	7. Other special conditions (e.g. nephritis, and other systemic disorders)	
	**Symptom assessment:** 8. NIH-CPSI total pain score > 4 to exclude men with chronic pelvic pain	8. NIH-CPSI total pain score < 10 to exclude patients without significant prostatitis-like pain
	**EPS examination:**	
	9. EPS sample can not be obtained for enough volume	
	10. EPS WBC/hpf ≥ 10 in EPS to exclude IV type prostatitis	10. EPS WBC/hpf ≤ 10 in EPS to exclude IIIB type prostatitis
	11. V1/V2/EPS/V3 bacterial culture positive to exclude urogenital tract infection (including I and II type prostatitis)	
	12. Positive examination of ureaplasma urealyticum, chlamydia trachomatis, neisseria gonorrhoeae and Mycoplasma in EPS	

Grey: common criteria for both healthy men and patients.

### Study subgroups

**Figure 2:**
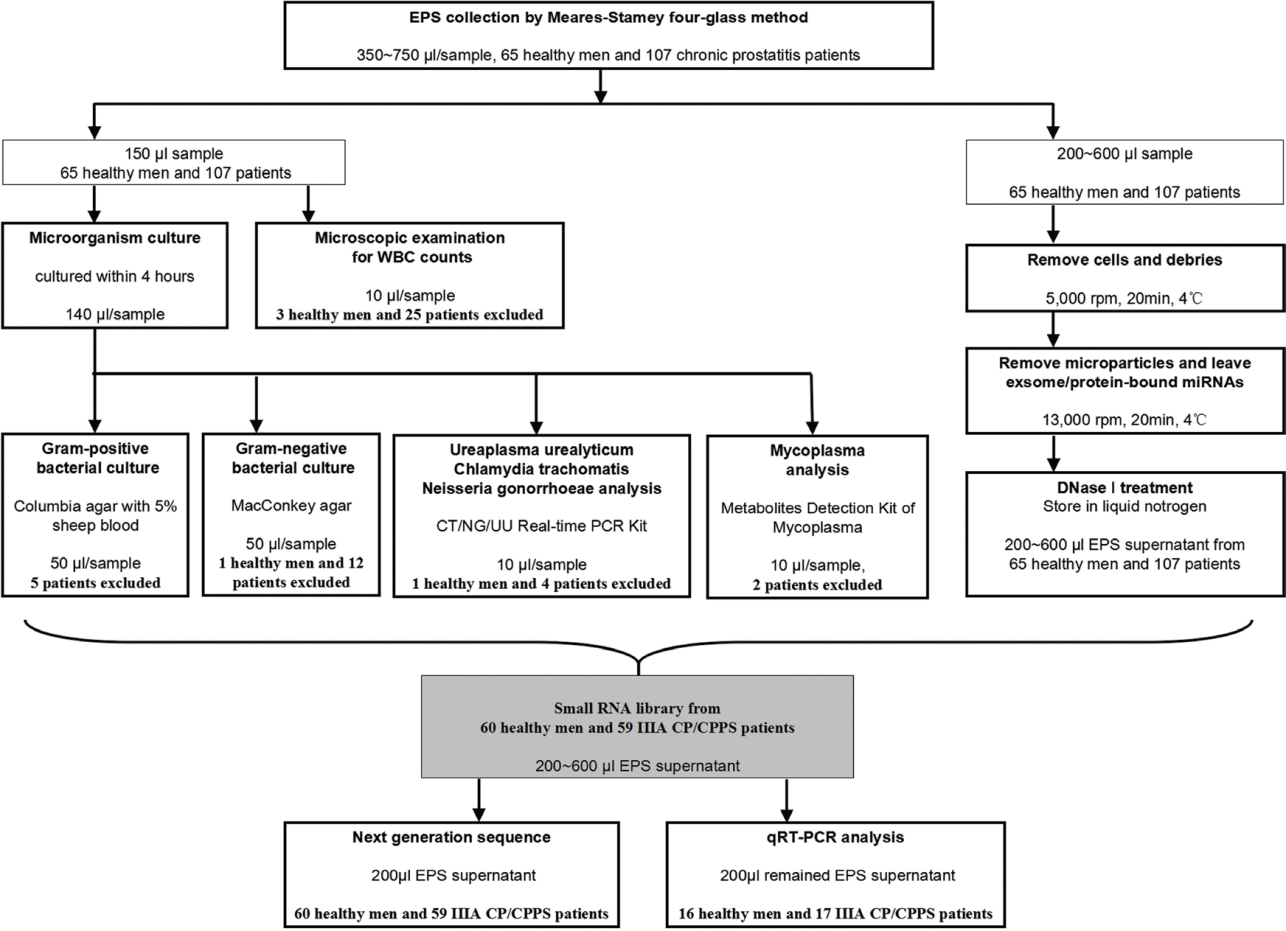
Flow diagram of EPS-sample collection and examination Each EPS sample was divided into 3 segments. First, 10 ul EPS were examined using a microscope for counting WBCs in EPS. Second, 140μl of every sample was used for microorganism test for excluding infection. Third, in every subgroup, 200 μl treated samples from every patient was pooled, and analyzed by Solexa sequence for identifying a comprehensive expression profile of EPS-miRNAs. Finally, if the volume of remaining supernatant was enough, 200 μl was used for quantifying up-regulated EPS-miRNAs.

As shown in Figure 1, Figure 2 and Table 1, 60 healthy men (designated as healthy group or H group) and 59 IIIA CP/CPPS patients (designated as patient group or P group) were ultimately selected for further study in a series of primary screen, symptom assessment, as well as EPS microscopy examination and culture. Then healthy men or IIIA CP/CPPS patients were randomly divided into 3 healthy subgroups (H1, H2 and H3) or 3 patient subgroups (P1, P2 and P3). H1 and H2 subgroups were utilized to explore the EPS-miRNA expression profile and screen stably abundant miRNAs in normal EPS. P1 and P2 subgroups were applied for screening stably abundant EPS-miRNAs in IIIA CP/CPPS patients. To identify differentially expressed EPS-miRNAs between healthy men and IIIA CP/CPPS patients, EPS-miRNA expression profiles were sequenced in P3 subgroup by comparing to H3 subgroup (Figure 1). Demographic and clinical characteristics of included participants in study subgroups are shown in Table 2.

**Table 2:** Demographic and clinical characteristics of included participants

Variables	Health Group			Disease Group			Comparison Group		
	Subgroup		*P*	Subgroup		*P*	Subgroup		*P*
	H1	H2		P1	P2		H3	P3	
Patients (*n*)	20	20		20	19		20	20	
Mean age (year)	31.0 ± 7.5	29.9 ± 7.4	0.64	30.9 ± 8.8	31.5 ± 8.3	0.82	28.1 ± 7.0	30.8 ± 8.0	0.25
Body mass index (kg/m^2^)	22.2 ± 1.7	21.8 ± 1.7	0.38	22.3 ± 1.6	21.7 ± 1.7	0.23	22.3 ± 1.7	21.7 ± 1.7	0.34
Marital status (*n*)									
Living with a partner	13 (65%)	12 (60%)	0.74	11 (55%)	13 (68%)	0.39	12 (60%)	13 (65%)	0.74
Single	7 (35%)	8 (40%)		9 (45%)	6 (32%)		8 (40%)	7 (35%)	
Employment status (*n*)									
Employed	15 (75%)	14 (70%)	0.72	19 (95%)	13 (68%)	0.03	19 (95%)	14 (70%)	0.04
Unemployed	5 (25%)	6 (30%)		1 (5%)	6 (32%)		1 (5%)	6 (30%)	
Sexual activity ^a^									
≤ 2 times weekly	12 (60%)	10 (50%)	0.53	13 (65%)	11 (58%)	0.65	13 (65%)	12 (60%)	0.11
≥3 times weekly	8 (40%)	10 (50%)		7 (35%)	8 (42%)		7 (35%)	8 (40%)	
Duration of pain (months)	0	0	1.00	6.3 ± 2.0	6.4 ± 2.2	0.86	0	5.9 ± 2.2	0.00
Total NIH-CPSI score	3.8 ± 2.5	4.3 ± 2.3	0.51	30.0 ± 4.2	26.9 ± 4.8	0.04	4.9 ± 1.8	30.7 ± 5.0	0.00
Total pain score	1.7 ± 1.5	2.0 ± 1.4	0.52	16.8 ± 2.6	15.3 ± 2.8	0.10	2.7 ± 1.5	16.7 ± 2.6	0.00
Total urination score	1.0 ± 0.9	1.2 ± 0.9	0.49	6.1 ± 2.3	4.4 ± 1.6	0.01	0.8 ± 0.6	5.8 ± 2.6	0.00
Quality of life score	1.2 ± 1.1	1.2 ± 1.1	1.00	7.1 ± 2.8	7.2 ± 2.7	0.86	1.4 ± 1.1	8.2 ± 2.9	0.00
Pain area									
Perineum	3 (15%)	2 (10%)	0.63	16 (80%)	16 (84%)	0.73	6 (30%)	17 (85%)	0.00
Testicles	2 (10%)	2 (10%)	1.00	15 (75%)	10 (53%)	0.15	4 (20%)	13 (65%)	0.00
Penis	5 (25%)	0 (0%)	0.02	14 (70%)	8 (42%)	0.08	5 (25%)	13 (65%)	0.01
Pubic	2 (10%)	1 (5%)	0.55	13 (65%)	8 (42%)	0.15	3 (15%)	11 (55%)	0.01
Pain during urination	2 (10%)	4 (20%)	0.38	17 (85%)	11 (58%)	0.06	2 (10%)	15 (75%)	0.00
Pain during ejaculation	0 (0%)	5 (25%)	0.02	17 (85%)	13 (68%)	0.22	2 (10%)	14 (70%)	0.00
IIEF-5 score ^b^	20.9 ± 4.6	20.5 ± 3.1	0.72	18.5 ± 4.3	16.7 ± 5.0	0.25	22.3 ± 2.3	15.7 ± 5.6	0.00
Perceived Stress Scale	14.6 ± 8.3	12.9 ± 8.4	0.51	20.0 ± 11.1	22.2 ± 10.5	0.54	12.5 ± 7.5	20.4 ± 9.8	0.00
Abstinent days ^c^	7.2 ± 2.5	6.5 ± 2.5	0.38	6.0 ± 2.4	6.6 ± 2.6	0.44	6.1 ± 2.0	6.2 ± 2.3	0.94
Mean EPS volume (ul)									
≤ 500	8 (40%)	8 (40%)	1.00	8 (40%)	11 (58%)	0.26	11 (55%)	10 (50%)	0.75
≥ 500	12 (60%)	12 (60%)		12 (60%)	8 (42%)		9 (45%)	10 (50%)	
WBC/HP in EPS ^d^									
I grade	20 (100%)	20 (100%)	1.00	0	0	0.07	20 (100%)	0	0
II grade	0	0		9 (45%)	14 (74%)		0	13 (65%)	
III grade	0	0		11 (55%)	5 (26%)		0	7 (35%)	

^a^ : Sexual activity in previous 6 months. ^b^ : The score of International Index of Erectile Function 5. ^c^ : Abstinent days before EPS collection. ^d^ : WBC count in EPS was classified into three grades: I grade: < 10/high power field (HP), II grade: 10∼20/HP, III grade: >20/HP.

### Follow-up group

21 previous patients were further enrolled in follow-up studies, and some of above differential expressed EPS-miRNAs were also assessed in patient-matched EPS samples collected at different time (pain score ≥ 10 vs pain score ≤ 4) in this self-control cohort (Figure 1). All follow-up patients received a standard treatment, including alpha-blockers (tamsulosin), phytotherapy (cernilton), physical therapy (electromagnetic therapy and hip bath), and adjustment of life style. Symptoms improvements were assessed throughout the whole follow-up period. In addition, the pain assessment and EPS collection in follow-up patients were carried out 1 week post-treatment withdrawal to ensure accurately record EPS-miRNAs profile changes caused by a relieved pain symptom of the prostate, rather than temporary changes stimulated by medication or/and physical therapy.

### EPS-miRNA expression profile in healthy men

**Figure 3:**
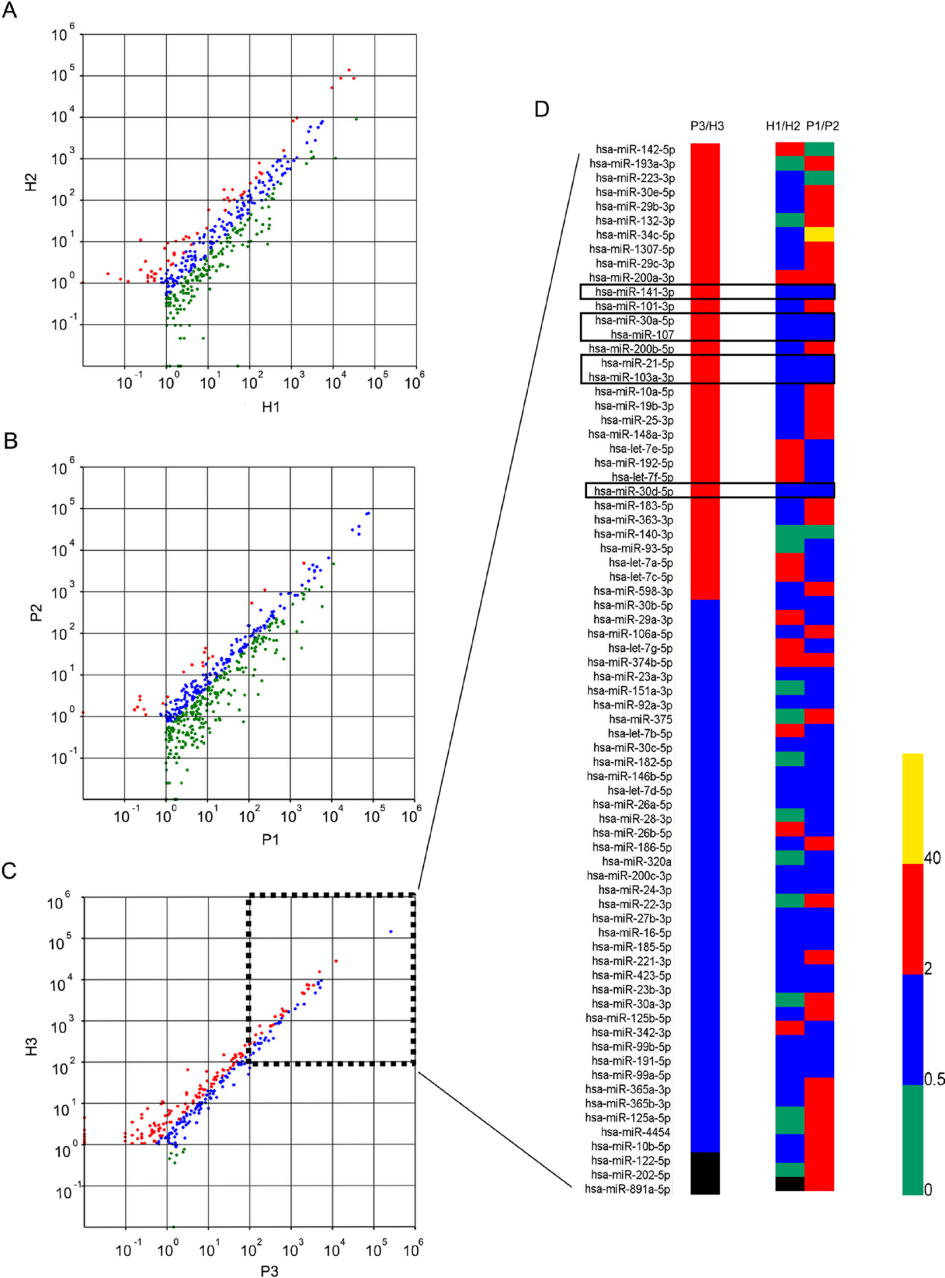
Scatter plot graph and heat map for EPS-miRNAs according to their normalized counts of high-throughput sequencing Every EPS-miRNA was showed with different color according to the ratio of normalized counts between two subgroups. Red (up-regulated), green (down-regulated), blue (equaled), and black (undetected). (**A**) Differential expression profile of normal EPS-miRNAs in bio-duplication subgroups (H1 and H2). 77 (H1) and 83 (H2) miRNAs were detected with normalized counts > 100. 66 EPS-miRNAs were simultaneously identified with normalized counts >100 in both healthy subgroups, and these miRNAs were considered as abundant EPS-miRNAs in healthy men. (**B**) Differential expression profile of EPS-miRNAs of IIIA CP/CPPS patients in bio-duplication subgroups (P1 and P2). 106 (P1) and 71 (P2) miRNAs were detected with normalized counts >100. 70 abundant EPS-miRNAs were identified in IIIA CP/CPPS patients. (**C**) Differentially expression profile of dysregulated EPS-miRNAs in P3 and H3 subgroups. Changed fold ranged from 0 to 2 was considered as being equally expressed (blue plot). In total, 22 abundant EPS-miRNAs (normalization counts >100) was up-regulated with change-fold >2 (red plots). (**D**) Change-folds of all abundant EPS-miRNAs from P3/H3, H1/H2, and P1/P2 subgroups. Results indicated 22 abundant EPS-miRNAs were up-regulated in P3 subgroup compared to H3 subgroup with more than 2 folds. Meanwhile, miR-21-5p, miR-30a-5p, miR-30d-5p, miR-103a-3p, miR-107, and mir-141-3p were stably expressed not only in the patient group but also in the health group.

To explore EPS-miRNA expression profile in healthy men, Solexa sequencing was employed to detect comparably small-RNAs reads between H1 and H2 subgroups aiming to provide a screen background of biological duplication. A total of 21,302,802 (84.5%) and 24,612,021 (83.3%) clean reads were obtained from H1 and H2 respectively, including 808 (H1) and 1052 (H2) miRNAs with raw counts ≥ 1, 161 (H1) and 191 (H2) miRNAs with normalized counts > 10, as well as 77 (H1) and 83 (H2) miRNAs with normalized counts > 100 (Figure 3A).

In fact, 66 EPS-miRNAs were simultaneously identified with normalized counts >100 in both healthy subgroups, and thus these miRNAs were considered as abundant EPS-miRNAs in healthy men. Results showed that most of these abundant EPS-miRNAs (95.5%) were in the form of single-arm (5p or 3p). After analyzing the ratio of normalized counts, 41 miRNAs were finally considered as the stably abundant EPS-miRNA in healthy men (Table 3). Meanwhile, the top 10 EPS-miRNAs of H1 and H2 subgroups were also listed in Table 3. Among them, 7 EPS-miRNAs (let-7a-5p, let-7b-5p, let-7c-5p, let-7f-5p, miR-21-5p, miR-375, and miR-103a-3p) were consistently enriched in EPS samples from different healthy control subgroups.

**Table 3:** The expression profile of EPS-miRNAs in health group and disease group

Stably abundant EPS-miRNAs							
Health Group				Disease Group			
MiRNA	H1	H2	H2/H1 Fold	MiRNA	P1	P2	P2/P1 Fold
**let-7d-5p**	662.26	363.77	0.55	**let-7a-5p**	77578.94	74867.08	0.97
**let-7i-5p**	194.90	142.90	0.73	**let-7b-5p**	70649.18	72327.38	1.02
**miR-101-3p**	565.28	812.94	1.44	**let-7c-5p**	32056.15	30586.95	0.95
**miR-103a-3p**	7008.23	5257.84	0.75	**let-7d-5p**	986.45	911.52	0.92
**miR-106a-5p**	451.21	715.18	1.59	**let-7e-5p**	1931.48	1411.38	0.73
**miR-106b-5p**	267.01	170.85	0.64	**let-7f-5p**	46791.44	37261.10	0.80
**miR-107**	4445.80	2609.13	0.59	**let-7g-5p**	2949.97	3431.39	1.16
**miR-10b-5p**	227.06	234.15	1.03	**let-7i-5p**	248.84	151.46	0.61
**miR-146b-5p**	148.62	183.89	1.24	**miR-103a-3p**	8726.56	6389.46	0.73
**miR-148a-3p**	1039.77	1387.57	1.33	**miR-107**	3901.75	3193.78	0.82
**miR-16-5p**	465.34	274.54	0.59	**miR-128-3p**	132.89	101.10	0.76
**miR-17-5p**	155.85	211.16	1.35	**miR-141-3p**	143.37	93.19	0.65
**miR-185-5p**	271.23	470.46	1.73	**miR-146b-5p**	401.32	327.34	0.82
**miR-191-5p**	2368.42	2393.83	1.01	**miR-151a-5p**	154.21	110.94	0.72
**miR-19b-3p**	185.37	286.77	1.55	**miR-152-3p**	191.48	111.11	0.58
**miR-200b-5p**	136.13	266.78	1.96	**miR-16-5p**	377.46	306.53	0.81
**miR-200c-3p**	838.58	469.00	0.56	**miR-185-5p**	605.11	860.33	1.42
**miR-21-5p**	7895.49	5713.39	0.72	**miR-191-5p**	3585.84	4295.99	1.20
**miR-23a-3p**	524.39	452.66	0.86	**miR-192-5p**	350.97	252.88	0.72
**miR-23b-3p**	294.70	171.79	0.58	**miR-193a-5p**	277.84	191.10	0.69
**miR-24-3p**	412.39	619.53	1.50	**miR-193b-3p**	244.93	130.17	0.53
**miR-25-3p**	565.79	966.97	1.71	**miR-200b-3p**	234.24	150.10	0.64
**miR-26a-5p**	1139.43	704.41	0.62	**miR-200c-3p**	929.41	904.72	0.97
**miR-29a-3p**	5751.36	2919.51	0.51	**miR-203a**	103.77	150.60	1.45
**miR-29b-3p**	406.52	303.19	0.75	**miR-21-5p**	11336.04	6586.28	0.58
**miR-29c-3p**	5565.32	4447.91	0.80	**miR-23a-3p**	1029.99	913.55	0.89
**miR-30a-5p**	672.17	591.82	0.88	**miR-23b-3p**	401.98	231.74	0.58
**miR-30b-5p**	906.03	551.36	0.61	**miR-24-3p**	197.48	359.90	1.82
**miR-30c-5p**	356.20	328.70	0.92	**miR-26a-5p**	2804.81	1775.80	0.63
**miR-30d-5p**	823.51	846.46	1.03	**miR-26b-5p**	3901.57	2998.89	0.77
**miR-30e-5p**	132.89	104.22	0.78	**miR-27a-3p**	136.71	118.82	0.87
**miR-331-3p**	112.99	110.03	0.97	**miR-29a-3p**	3944.10	2087.09	0.53
**miR-363-3p**	885.33	1162.40	1.31	**miR-30a-5p**	699.35	419.27	0.60
**miR-365a-3p**	231.99	377.05	1.63	**miR-30b-5p**	739.22	396.40	0.54
**miR-365b-3p**	231.99	377.05	1.63	**miR-30c-5p**	391.62	262.67	0.67
**miR-423-5p**	2760.53	3811.92	1.38	**miR-30d-5p**	1312.97	800.01	0.61
**miR-574-3p**	297.33	331.06	1.11	**miR-320a**	5499.88	3203.94	0.58
**miR-598-3p**	264.28	210.18	0.80	**miR-342-3p**	309.05	216.90	0.70
**miR-92a-3p**	391.45	625.79	1.60	**miR-375**	46550.06	23526.90	0.51
**miR-99a-5p**	907.30	492.77	0.54	**miR-423-5p**	4458.66	3858.82	0.87
**miR-99b-5p**	295.22	298.59	1.01	**miR-574-3p**	196.41	134.56	0.69
				**miR-744-5p**	168.31	126.65	0.75
				**miR-92a-3p**	1527.38	818.61	0.54
				**miR-93-5p**	359.04	264.68	0.74
				**miR-99a-5p**	584.47	441.94	0.76
				**miR-99b-5p**	368.74	325.30	0.88

The top 10 EPS-miRNAs				
Rank	Health Group		Disease Group	
	H1 subgroup	H2 subgroup	P1 subgroup	P2 subgroup
**1**	let-7a-5p	miR-375	let-7a-5p	let-7a-5p
	137505	36790	77578	74867
**2**	let-7f-5p	let-7b-5p	let-7b-5p	let-7b-5p
	87358	31665	70649	72327
**3**	let-7b-5p	let-7a-5p	let-7f-5p	let-7f-5p
	84877	24345	46791	37261
**4**	let-7c-5p	let-7f-5p	miR-375	let-7c-5p
	51008	15340	46550	30586
**5**	let-7g-5p	miR-193a-3p	let-7c-5p	miR-375
	9448	11574	32056	23526
**6**	miR-375	let-7c-5p	miR-21-5p	miR-21-5p
	8782	9505	11336	6586
**7**	miR-26b-5p	miR-21-5p	miR-103a-3p	miR-103a-3p
	8126	5713	8726	6389
**8**	miR-21-5p	miR-103a-3p	miR-193a-3p	miR-140-3p
	7895	5257	5976	4804
**9**	miR-103a-3p	miR-29c-3p	miR-29c-3p	miR-191-5p
	7008	4447	5866	4295
**10**	miR-29a-3p	miR-423-5p	miR-320a	miR-423-5p
	5751	3811	5499	3858

### EPS-miRNA expression profile in IIIACP/CPPS patients

By Solexa sequencing, 33,486,743 (92.9%) and 39,833,420 (83.1%) clean reads were obtained from P1 and P2 sRNA cDNA libraries, respectively. A total of 1337 (P1) and 937 (P2) miRNAs were expressed with raw counts > 1 (Figure 3B). The common top-10 EPS-miRNAs in both patient subgroups were let-7a-5p, let-7b-5p, let-7c-5p, let-7f-5p, miR-21-5p, miR-375, and miR-103a-3p, which was consistent with the expression spectrum in healthy men (Table 3). Meanwhile, 106 (7.9%) and 71 (7.6%) miRNAs with normalized counts > 100 were detected in P1 and P2 subgroup, respectively. Finally, 70 abundant EPS-miRNAs were identified in IIIA CP/CPPS patients, and 43 miRNAs were considered as stably abundant EPS-miRNA in IIIA CP/CPPS patients (Table 3).

### EPS-miRNAs associated with IIIA CP/CPPS

#### Discovery phase

To further identify differential EPS-miRNAs in IIIA CP/CPPS patients, Solexa sequencing was performed comparably in pool-samples between P3 and H3 subgroups. Results showed 54 (13.0%) miRNA were expressed in P3 subgroup with normalized counts > 100. Meanwhile, a total of 283 (68.2%) EPS-miRNAs were increased with change-fold > 1 in P3 subgroup compared to H3 subgroup, and 148 (35.7%) EPS-miRNAs were increased with change-fold > 2 (Figure 3C). Among them, the normalized counts of 22 miRNAs were more than 100 (Table 4). Importantly, miR-21-5p, miR-30a-5p, miR-30d-5p, miR-103a-3p, miR-107, and mir-141-3p were the EPS-miRNAs which were not only stably expressed in patients but also in healthy men (Figure 3D and Table 5). These 6 stably differential EPS-miRNAs were included to further identify in testing phage.

**Table 4:** Abundant EPS-miRNAs differentially expressed in IIIA CP/CPPS patients compared to healthy men

EPS-MiRNA	Comparison Group			Health Group H2/H1 Fold	Disease Group P2/P1 Fold
	P3	H3	P3/H3 Fold		
miR-30e-5p	729.34	178.10	4.10	0.78	0.21
miR-200a-3p	449.09	142.90	3.14	0.43	0.44
miR-141-3p	14960.17	4929.89	3.03	0.75	0.65
miR-101-3p	1872.78	619.24	3.02	1.44	0.47
miR-30a-5p	7324.17	2460.57	2.98	0.88	0.60
miR-107	1226.86	421.50	2.91	0.59	0.82
miR-98-5p	274.63	103.76	2.65	0.14	0.44
miR-21-5p	7158.38	2744.56	2.61	0.72	0.58
miR-148b-3p	499.68	196.43	2.54	0.59	0.49
miR-103a-3p	1643.27	666.45	2.47	0.75	0.73
miR-10a-5p	4630.96	1899.24	2.44	1.34	0.07
miR-19b-3p	1386.08	577.76	2.40	1.55	0.48
miR-25-3p	730.49	324.84	2.25	1.71	0.49
miR-148a-3p	27642.08	12371.25	2.23	1.33	0.39
miR-203a	296.09	133.90	2.21	0.49	1.45
miR-192-5p	733.45	336.26	2.18	0.46	0.72
let-7f-5p	5786.28	2681.02	2.16	0.18	0.80
miR-30d-5p	1642.32	774.87	2.12	1.03	0.61
miR-183-5p	4414.73	2089.05	2.11	1.20	0.47
miR-363-3p	781.63	372.88	2.10	1.31	0.42
let-7a-5p	7130.00	3514.19	2.03	0.18	0.97
let-7c-5p	1292.39	638.20	2.03	0.19	0.95

**Table 5:** Demographics and clinical characters of participants in testing phage

	Health men			IIIA CP/CPPS patients			*P* value
Subgroup	H1	H2	H3	P1	P2	P3	
Participants (*n*)	12	12	9	10	12	8	
Total participants (*n*)	33			30			
Age (year)	31.6 ± 7.4			30.2 ± 8.1			0.47
Body mass index (kg/m2)	22.1 ± 1.7			21.9 ± 1.5			
Employment status							
Employed	8 (24%)			7 (23%)			0.93
Unemployed	25 (76%)			23 (77%)			
Marital status							
Living with a partner	8 (24%)			13 (43%)			0.11
Single	25 (76%)			17 (57%)			
Sexual activity ^a^							
≤ 2 weekly	19 (58%)			17 (57%)			0.94
≥3 times weekly	14 (42%)			13 (43%)			
Duration of Pain (month)	0			6.0 ± 2.2			0
Total NIH-CPSI score	4.6 ± 2.3			28.9 ± 5.2			0
Total pain score	2.2 ± 1.5			16.5 ± 2.7			0
Total urination score	1.1 ± 0.9			5.4 ± 2.5			0
Quality of life score	1.3 ± 1.2			7.0 ± 2.7			0
Pain area							
Perineum	6 (18%)			25 (83%)			0.00
Testicles	4 (12%)			19 (63%)			0.00
Penis	5 (15%)			20 (67%)			0.00
Pubic	5 (15%)			18 (60%)			0.00
Pain during urination	5 (15%)			20 (67%)			0.00
Pain during ejaculation	4 (12%)			22 (73%)			0.00
IIEF-5 score ^b^	21.9 ± 2.6			17.8 ± 5.2			0.00
Perceived Stress Scale	13.1 ± 9.2			22.5 ± 9.8			0.00
Abstinent Days ^c^	7.2 ± 2.6			6.5 ± 2.2			0.21
WBC/HP in EPS ^d^							0
I grade	33 (100%)			0			
II grade	0			17 (57%)			
III grade	0			13 (43%)			

^a^ : Sexual activity in previous 6 months. ^b^ : The score of International Index of Erectile Function 5. ^c^ : Abstinent days before EPS collection. ^d^ : WBC count in EPS was classified into three grades: I grade: < 10/high power field (HP), II grade: 10∼20/HP, III grade: > 20/HP.

### Testing phase

**Figure 4:**
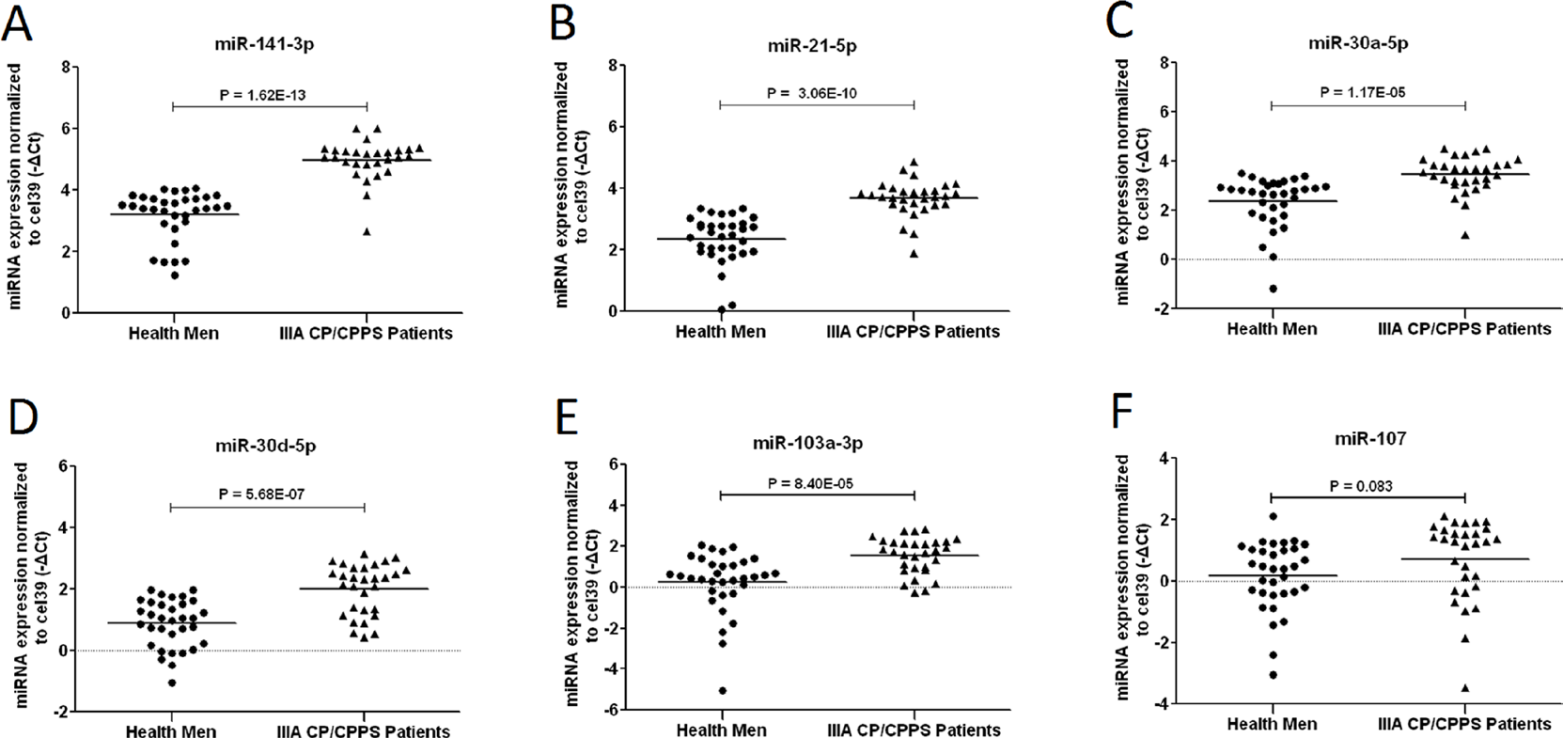
Identification of screened 6 EPS-miRNA in testing phage Raw -ΔCt value normalized to cel-mir-39 (y-axis) of 6 up-regulated EPS-miRNAs (miR-141-3p, miR-30a-5p, miR-21-5p, miR-107, miR-103a-3p, and miR-30d-5p) were compared between 30 IIIA CP/CPPS patients (triangle) and 33 healthy men (rotundity). Among them, miR-141-3p was significantly increased with 3.4 folds (Figure 3A). And other 4 miRNAs were also found to be up-regulated in patients (2.5-fold for miR-21-5p, 2.14-fold for miR-30a-5p, 2.19-fold for miR-30d-5p, and 2.48-fold for miR-103a-3p, Figure 3B∼3E). However, no significant difference was detected for miR-107 between two groups (*p* = 0.08). Fold value = 2^[-(mean ΔCt of patients - mean ΔCt of controls)]^.

To validate the results of Solexa Sequence, Taqman qRT–PCR assays were performed for 6 screened miRNAs in all remaining EPS-samples individually (33 healthy men and 30 IIIA CP/CPPS patients). Demographic and clinical characteristics of participants in testing phase are listed in Table 5. Compared to healthy men, miR-141-3p was significantly increased with 3.4-fold, and 4 miRNAs (miR-21-5p, miR-30a-5p, miR-30d-5p: and miR-103a-3p) were also remarkably up-regulated with more than 2-fold in IIIA CP/CPPS patients (Figure 4). The top 3 up-regulated EPS-miRNAs (miR-141-3p, miR-21-5p and miR-103a-3p) were further included into validation phase.

### Validation phase

**Figure 5:**
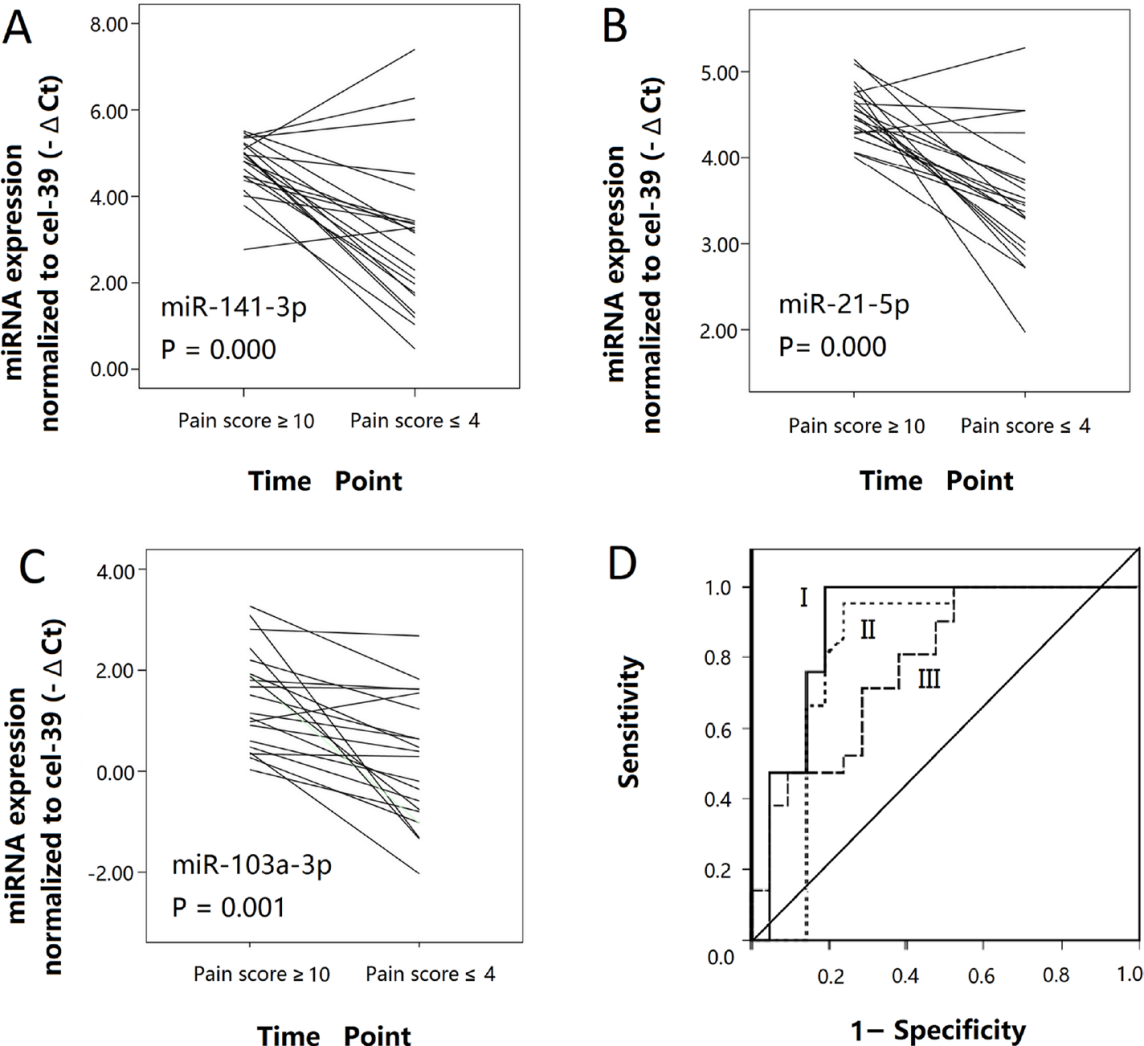
Confirmation of top 3 dysregulated EPS-miRNA in validation phage (**A**–**C**) Line graphs were employed for analyzing the changed normalized -ΔCt value of miR-141-3p, miR-21-5p, and miR-103a-3p in 21 follow-up patients. –ΔCt values of top 3 dysregulated EPS-miRNA were decreased when pain symptom was relieved. Every line represents one follow-up patient. (**D**) Performance of miR-21-5p (I), miR-141-3p (II), and miR-103a-3p (III) in discriminating IIIA CP/CPPS patients with significant pain symptom in follow-up cohort. Data of ROC curve was derived from the normalized -ΔCt value. The comparison was acted between pain score ≥ 10 and pain score ≤ 4. ROC curve analyses indicated that miR-21-5p showed the highest diagnostic accuracy with an area under the ROC curve (AUC) of 0.891 (95%CI: 0.669–0.972).

Levels of miR-141-3p, miR-21-5p and miR-103a-3p were measured traceably in 21 patients from P1, P2, and P3 subgroups with mean follow-up time of 8.8 ± 5.5 (1∼21) months. Compared to the EPS sample collected at the time diagnosed as chronic prostatitis with significant pain (pain score > 10), the EPS sample collected at the time with relieved symptom (pain score < 4) possessed decreased levels of these 3 miRNAs(miR-141-3p: 5.54-fold, miR-21-5p: 1.7-fold, and miR-103a-3p: 3.2-fold, mean Fold = mean of 2^(-ΔΔCt of every patient)^) in most of subjects in this self-control cohort (Figure 5A–5C).Interestingly, ROC curve analysis indicated miR-21-5p possessed the highest classify accuracy with an area under the ROC curve (AUC) of 0.891 (95%CI: 0.669–0.972) (Figure 5D). At the cutoff value of 1.81, the optimal sensitivity and specificity were 100.0% and 81.0%, respectively.

## DISCUSSION

MiRNAs are emerging as a new layer of regulators in human diseases, while it remains unknown whether miRNAs can be secreted into EPS. In current study, we performed a comprehensive expression profile analysis of secreted miRNA in human EPS by using high-throughput sequencing, and identified 41 and 43 predominant miRNAs in health men and IIIA CP/CPPS patients respectively (Table 3). Therefore, our work provided a proof-of-concept that secreted miRNAs could abundantly and stably exist in prostatic fluid. Interesting, let-7a-5p, let-7b-5p, let-7c-5p, let-7f-5p, miR-21-5p, miR-375 and miR-103a-3p were consistently ranked among the top 10 EPS-miRNAs in both healthy men and patients. In support, many of these stably abundant miRNAs have been linked to fertilization in mammals. For instance, Oliver Rando and colleagues found that paternal diet can influence offspring phenotype via altered levels of small RNAs in mouse sperm, including tRNA fragments and let-7 [20]. Moreover, intracellular miR-21 in mouse sperm was found to regulate the self-renewal of spermatogonial stem cells [21], and human sperm-originated miR-21 and miR-375 were found to be implicated in embryo quality *in vitro* fertilization [22]. Considering the crucial role of prostate fluid in capacitation, acrosome reaction and motility of sperm [1, 5], our work shed a new light on the biological function of prostate fluid, implying that EPS-miRNAs may serve as a new player in andrology and male urology.

To greatly minimize potential interference caused by possible inconsistency in pathological process between IIIA and IIIB CP/CPPS [19, 23], IIIB CP/CPPS patients were excluded in current study. In addition, some articles showed aberrant expression of specific miRNAs was associated with certain male reproductive dysfunctions [22, 24, 25]. Therefore, we also excluded patients with infertility to reduce potential interference by idiopathic etiology. Under the design with a “prostate centric” approach, we found that most abundant EPS-miRNAs were up-regulated in IIIA CP/CPPS, and 6 abundant miRNAs (miR-21-5p, miR-30a-5p, miR-30d-5p, miR-103a-3p, miR-107 and miR-141-3p) were significantly increased in IIIA CP/CPPS patients. There were two potential resources that would mainly contribute to these secretory EPS-miRNAs: 1. intrinsic miRNAs secreted by prostate, which could be abnormally expressed under damage or stress in prostate epithelial cells, 2. miRNAs secreted by other types of cells, which could be produced during pathological process of IIIA CP/CPPS, such as inflammatory leukocytes. Recently, many studies have revealed an important role of secretory miRNAs in different types of human diseases. For instance, an increased circulating miR-21-5p level in serum was reported to be associated with kidney fibrosis [26]. MiR-888 in EPS-urine was found to be preferentially elevated in high-grade prostate cancer patients [27] And Melman found that circulating miR-30d-5p was associated with response to cardiac resynchronization therapy in heart failure and regulated cardiomyocyte apoptosis [28]. Thus, our results provided the first evidence that EPS-miRNAs could be a novel type of bio-molecules involving in the progression of IIIA CP/CPPS.

Although a great effort has been made in the study of prostatitis, a reliable characteristic to diagnose prostatitis is still a big challenge [29]. Our follow-up studies showed that abnormally high levels miR-141-3p, miR-21-5p and miR-103a-3p was correlated with increased pain score in IIIA CP/CPPS patients. Importantly, ROC analysis showed that miR-21-5p possessed a relatively high classify-accuracy for IIIA CP/CPPS patients with significant pelvic pain. In addition, some recent studies reported that urinary microbiomes from patients with CP/CPPS have a significantly higher alpha (phylogenetic) diversity and higher counts of Clostridia [30], as well as increased levels of urine prostatic exosomal protein than healthy controls [31]. Taken together, these evidences suggested that EPS-miRNAs may offer a new approach towards a better diagnosis and outcome monitoring of prostatitis, with combination of proteomic analyses [3], nucleic acid analyzing [2] and microorganism genomic sequencing [32].

In summary, we demonstrated that miRNAs abundantly existed in EPS by next generation sequencing. Some of EPS-miRNAs (e.g. miR-141-3p, miR-21-5p and miR-103-3p) were differentially expressed between healthy men and patients with IIIA CP/CPPS. Of interest, miR-21-5p was found to be well associated with increased pain score in patients with IIIA CP/CPPS in follow-up studies. Therefore, our studies provided a proof-of-concept of EPS-miRNAs and its clinical relevance to prostatitis.

## MATERIALS AND METHODS

### Inclusion and exclusion criteria for participants

Health men recruited from our health-check center and outpatients diagnosed with chronic prostatitis within 1 year were involved in this study. Subjects with age 18 to 45 were chosen to minimize individually developmental variation of prostate, potential risk of hyperplasia or prostate cancer. Then health men and patients were further excluded by following common criteria: (a) urine-culture/urethral-swab test/EPS-culture was positive, (b) previous surgery and permanent medication of urogenital tract (chronic prostatitis was not included), (c) urogenital congenital malformation and malignancy, (d) lithiasis, (e) neurogenic disease of the bladder, (f) infertility, and (g) EPS sample cannot be obtained for enough volume. Other health men were excluded by following additional criteria: (a) total pain score > 4, (b) WBC/hpf ≥ 10 in EPS (IV type prostatitis). For our clinical screen, the evaluation of pain was assessed by using NIH-CPSI (NIH-Chronic Prostatitis Symptom Index). NIH-CPSI is a widely used 13-item questionnaire to assess symptom and quality of life in men with CP/CPPS (Chronic prostatitis/Chronic pelvic pain syndrome), and it has a total score range from 0 to 43, including three subscales addressing pain (score range 0–21), urinary symptoms (score range 0–10) and quality of life (score range 0–12) in the latest week. In clinic, CP/CPPS is diagnosed from a history of pain perceived in the region of the prostate (convincingly reproduced by prostate palpation), and absence of other lower urinary tract pathology for a minimum of 3 out of the past 6 months. Meanwhile, other patients were further excluded by following special criteria: (a) total pain score < 10, (b) WBC/hpf < 10 in EPS (IIIB CP/CPPS) (Detailed inclusion and exclusion criteria were showed in Table 1).

### EPS-sample collection and microscopic examination

EPS sample was individually collected from subject abstaining from sexual activity for at least 3 days. By four-glass method, individual EPS sample (350∼750 μl) from each subject was divided into 3 segments for microscopic examination (10 μl), microorganism test (140 μl) and Solexa sequencing (200 μl). If the volume of remaining supernatant was enough, 200 μl was stored for qRT-PCR (Figure 2). 10 μl EPS were placed on sterilized microscope slides covered with a cover slip and examined using a microscope. Counts were expressed as the number of WBCs per high power field (hpf). In general, WBC was counted at 400 × power (average of fields rounded to nearest whole number). Counts were expressed as the number of WBCs per hpf up to a total of 25. For fields with more than 25 WBC/hpc, the number was expressed as 25+.

### Bacterial culture for EPS samples

The cultures of all samples were performed in the department of clinical laboratory of our hospital. Briefly, 100 μl of each sample was utilized for common bacterial culture. EPS were cultured within 4 hours post-collection by spreading 50 μl of each specimen onto plates containing Columbia agar with 5% sheep blood at 37°C in a 5% CO2 enriched atmosphere for the detection of Gram-positive bacteria. Another 50 μl of EPS were cultured at 36°C on MacConkey agar to detect Gram-negative bacteria. All samples were examined for bacterial growth after 2∼5 days. If fastidious microorganisms were suspected to be present in the EPS, further culture would be carried out in different agar (e.g. ThayereMartin agar and vaginalis agar) or under different culture condition (e.g. anaerobic circumstance, with or without CO2) by using new collected samples again. In addition, the participants with suspicious infection in EPS were excluded during clinical screen.

### Examination on special infection in EPS

After EPS collection in urologic laboratory, 20∼30μl EPS of each participant was immediately used to examine the infection of mycoplasma (10 μl for detecting metabolites produced by the mycoplasma, Mycoplasma Detection Kit-QuickTest, Biotool, Houston, TX, US) and Ureaplasma urealyticum/Chlamydia trachomatis/Neisseria gonorrhoeae (10ul, CT/NG/UU Real-time PCR Kit, hybribio, Hongkong, China) following the manufacturer›s instructions.

### EPS samples for High-throughput sequencing and qRT-PCR

After microscopic and microbiological examination, remaining samples were centrifuged at 5,000 rpm for 20 min at 4°C to remove cell debris (including WBC). The supernatants were collected and centrifuged again at 13,000 rpm for 20 min at 4°C to remove other microparticles, leaving exosomes (including protein-bound miRNAs). DNase I (Takara, Dalian, China) treatment was performed to remove any contaminating DNA. All supernatants were stored in liquid nitrogen. To minimize individual variations, in every subgroup, 200 μl supernatant from every sample was pooled, and analyzed by Solexa sequence aiming to identify the comprehensive profile of EPS-miRNAs. Finally, if the volume of remaining supernatant was enough, 200 μl was used for quantifying up-regulated EPS-miRNAs by real-time PCR, and the cDNA libraries were stored.

### High-throughput sequencing

For each subgroup of healthy men or IIIA CP/CPPS patients, 200 μl EPS supernatant from each participant was pooled and analyzed to minimize individual variations. Briefly, total RNA of pooled EPS samples was isolated by miRCURY RNA isolation kit for biofluids (Exiqon, Woburn, US) following the manufacturer›s instructions. 5’ and 3’ adaptors were ligated to the small RNAs (10∼44 nt) after gel-extraction. Adaptor-ligated RNAs were subsequently transcribed into cDNA and amplified by PCR [14]. The resultant products were purified and subjected to Solexa sequencing (Beijing Genomics Institute, ShenZhen, China). The counts of miRNAs was normalized [normalization counts = (actual count/total count of clean reads) × 1,000,000], and only miRNAs with normalized counts >100 in both bio-duplication subgroups (H1 and H2, or P1 and P2) were considered as abundant EPS-miRNAs. Stable miRNAs were defined if their ratio of normalized counts in paired subgroups ranged from 0.5 to 2.0.

### Taqman-based quantitative real-time PCR (qRT-PCR)

For qRT-PCR, total RNA was individually extracted from each EPS sample. The first-strand miRNA-cDNA PCR template was generated from total RNA using the TaqMan^®^ MicroRNA Reverse Transcription Kit (Applied Biosystems), including an artificial RNA spike-in (cel-mir-39) as loading control [27, 33]. The cDNA was then used in PCR on a 7500HT real-time PCR instrument (Applied Biosystems). Triplicate samples, and inter-assay controls were used throughout. For each assay, the Ct (Cycle threshold) of miRNA was subtracted from the average cel-mir-39 Ct value to obtain a ΔCt value. The relative folds were calculated utilizing the 2^(-ΔCt)^ method.

### Statistical Analysis

Clinical characteristics were compared using x^2^ and *t*-test. Expression levels of miRNAs were analyzed employing *t*-test. Receiver operating characteristic (ROC) curve were established to evaluate the diagnostic value of EPS-miRNAs. In ROC analysis, -ΔCt normalized to cel-mir-39 was selected as the test variable for included miRNAs. *P* < 0.05 (2-tailed) was considered statistically significant. All analyses were performed by SPSS 17.0.

## Abbreviations

MiRNAs: microRNAs
EPS: expressed prostatic secretion
CP/CPPS: Chronic prostatitis/Chronic pelvic pain syndrome
ROC: receiver operating characteristic
AUC: area under ROC curve
hpf: high power field
WBC: white blood cell
qRT-PCR: Taqman-based quantitative real-time PCR
Ct: Cycle threshold
